# Supplementary material for: Transmitted HIV-1 is more virulent in heterosexual individuals than men-who-have-sex-with-men
Source: PLoS Pathog. 2022 Mar 10;18(3):e1010319. doi: 10.1371/journal.ppat.1010319 (PMC8912199; doi:10.1371/journal.ppat.1010319)
Supplement: S1 Table — Data from different regions in Europe show substantially higher subtype B percentage prevalence in MSM than HET (P < 0.001 in each study, unless specified). The sample sizes (n) are in parantheses. (PDF) [file ppat.1010319.s001.pdf]

S1 Table. Prevalence of HIV-1 subtype B in Europe. Data from different regions in Europe show substantially higher subtype B percentage prevalence in MSM than HET ($P < 0.001$ in each study, unless specified). The sample sizes (n) are in parantheses.

Region	Survey year(s)	Prevalence of subtype B (%)	
		MSM (n)	HET (n)
France [1]	2001<br>2006 – 07<br>2010 – 11	80.5 (113)<br>90.6 (223)<br>83.3 (311)	53.2 (186)<br>27.2 (202)<br>26.2 (263)
Italy [2]	2000 – 14	81.5 (1,824)	54.9 (1,573)
UK [3]	2002 – 10	$88.4^{‡}$ (21,321)	$13.3^{‡}$ (38,948)
Madrid, Spain [4]	2000 – 11	88.2 (340)	41.5 (369)
Comunidad Valenciana (CV), Spain [5]	2004 – 14	$92.0^{‡}$ (637)	$60.8^{‡}$ (204)
Europe [6]	2002 – 07	90.4 (2,084)	33.5 (1,501)
Canada* & Europe [7]	1998 – 10	$95.5^{‡}$ (5,570)	$38.7^{‡}$ (2,052)

$^{‡}P$ value not specified.
*15.9% patients were from Canada.

References

1. Descamps, D. *et al.* National sentinel surveillance of transmitted drug resistance in antiretroviral-naive chronically HIV-infected patients in France over a decade: 2001–2011. *J. Antimicrob. Chemother.* **68**, 2626–2631 (2013). URL https://doi.org/10.1093/jac/dkt238.
2. Fabeni, L. *et al.* Dynamics and phylogenetic relationships of HIV-1 transmitted drug resistance according to subtype in Italy over the years 2000–14. *J. Antimicrob. Chemother.* **72**, 2837–2845 (2017). URL https://doi.org/10.1093/jac/dkx231.
3. The UK Collaborative Group on HIV Drug Resistance. The increasing genetic diversity of HIV-1 in the UK, 2002–2010. *AIDS* **28**, 773–780 (2014). URL https://journals.lww.com/aidsonline/Fulltext/2014/03130/The_increasing_genetic_diversity_of_HIV_1_in_the.15.aspx.
4. Yebra, G. *et al.* Different trends of transmitted HIV-1 drug resistance in Madrid, Spain, among risk groups in the last decade. *Arch. Virol.* **159**, 1079–87 (2014). URL https://doi.org/10.1007/s00705-013-1933-y.
5. Patino-Galindo, J. A. *et al.* The molecular epidemiology of HIV-1 in the Comunidad Valenciana (Spain): analysis of transmission clusters. *Sci. Rep.* **7**, 11584 (2017). URL https://doi.org/10.1038/s41598-017-10286-1.
6. Frentz, D. *et al.* Patterns of transmitted HIV drug resistance in Europe vary by risk group. *PLoS ONE* **9**, e94495 (2014). URL https://doi.org/10.1371/journal.pone.0094495.
7. Klein, M. B. *et al.* The effects of HIV-1 subtype and ethnicity on the rate of CD4 cell count decline in patients naive to antiretroviral therapy: a Canadian-European collaborative retrospective cohort study. *CMAJ OPEN* **2**, E318–E329 (2014). URL https://doi.org/10.9778/cmajo.20140017.
