# Supplementary material for: Transmitted HIV-1 is more virulent in heterosexual individuals than men-who-have-sex-with-men
Source: PLoS Pathog. 2022 Mar 10;18(3):e1010319. doi: 10.1371/journal.ppat.1010319 (PMC8912199; doi:10.1371/journal.ppat.1010319)
Supplement: S2 Table — A recent review [21] of 130 published articles, together involving of 10,516 MSM and 6,759 HET individuals, has examined the prevalence of different subtypes in China, which is reproduced below. P values indicate significant differences in the prevalences of 3 subtypes. (PDF) [file ppat.1010319.s002.pdf]

S2 Table. Prevalence of HIV-1 subtypes in China. A recent review [1] of 130 published articles, together involving of 10,516 MSM and 6,759 HET individuals, has examined the prevalence of different subtypes in China, which is reproduced below. P values indicate significant differences in the prevalences of 3 subtypes.

Subtype	Prevalence (%) (95% CI)		
	MSM	HET	P value
CRF01_AE	51.28 (46.15 – 56.40)	38.78 (33.08 – 44.63)	< 0.01
CRF07_BC	19.98 (16.17 – 24.07)	14.88 (10.96 – 19.23)	0.083
CRF08_BC	0.00 (0.00 – 0.00)	9.81 (6.44 – 13.70)	< 0.01
B\B′	17.74 (12.78 – 23.26)	15.41 (11.15 – 20.16)	0.508
C	0.00 (0.00 – 0.00)	1.89 (0.81 – 3.28)	< 0.01
Others	1.56 (0.98 – 2.24)	2.39 (1.33 – 3.66)	0.2109

References

1. Yuan, R., Cheng, H., Chen, L. S., Zhang, X., and Wang, B. Prevalence of different HIV-1 subtypes in sexual transmission in China: a systematic review and meta-analysis. *Epidemiol. Infect.* **144**, 2144–2153 (2016). URL `https://doi.org/10.1017/S0950268816000212`.
