# Supplementary material for: Transmitted HIV-1 is more virulent in heterosexual individuals than men-who-have-sex-with-men
Source: PLoS Pathog. 2022 Mar 10;18(3):e1010319. doi: 10.1371/journal.ppat.1010319 (PMC8912199; doi:10.1371/journal.ppat.1010319)
Supplement: S3 Table — The sources of the studies, the periods of study, measurement times, and other details are mentioned. The USA and European studies report IQRs, whereas the CASCADE study provides 95% CIs. NA—not available. (PDF) [file ppat.1010319.s003.pdf]

S3 Table. Early median CD4 cell counts in infected adults from several large population studies*. The sources of the studies, the periods of study, measurement times, and other details are mentioned. The USA and European studies report IQRs, whereas the CASCADE study provides 95% CIs. NA - not available.

Region (Reference)	Time period	Measurement time	Risk group	CD4 counts ($cells/\mu L$)	Sample size (n)	Other details
USA‡ [1]	2006 – 15	Within 6 m.o. diagnosis	MSM	387 (227 – 555)	17,779	
			HET	308 (125 – 508)	8,310	
Europe [2]	2002 – 07	Within 6 m.o. diagnosis	MSM	435 (259 – 585)	2,084	$P < 10^{-3}$
			HET	280 (110 – 458)	1,501	
England & Wales [3]	1990 – 98	Within 6 m.o. diagnosis	MSM	331 (NA)**	6,213	$P < 10^{-3}$
			HET	230 (NA)**	2,637	
Europe & Australia SCs†† [4]	1979 – 00	Seroconversion	MSM (age<40)	621 (609 – 634)	2,941	$n = 5739$‡‡. 54.7% of n (MSM & hemophiliac men) only men. 20.4% of n women. 12.6% of n aged>40.
			MSM (age>40)	578 (556 – 601)		
			HET men (age<40)	576 (545 – 607)	889	
			HET men (age>40)	534 (501 – 569)		
			HET women (age<40)	623 (599 – 647)		
			HET women (age>40)	580 (548 – 612)		

*When many studies were available with overlapping durations and geographical locations, we chose data from the study with the larger sample size, (as in the case of New York city [1]).
‡The proportion of females was 22.2% and the age group 13-29 had 35.6% of $n = 28,162$ individuals.
**We chose the SDs from the Europe (2002-07) study (see Methods).
††We used the SDs corresponding to age < 40 years. For HET, we used the largest SD among HET men and women.
‡‡n includes MSM, HET, injection drug users, and hemophiliac men.

References

1. Robertson, M. M., Braunstein, S. L., Hoover, D. R., Li, S. & Nash, D. Estimates of the time from seroconversion to ART initiation among people newly diagnosed with HIV from 2006 to 2015, New York City. *Clin. Infect. Dis.* **71**, e308–e315 (2019). URL `https://doi.org/10.1093/cid/ciz1178`.

2. Frentz, D. *et al.* Patterns of transmitted HIV drug resistance in Europe vary by risk group. *PLoS ONE* **9**, e94495 (2014). URL `https://doi.org/10.1371/journal.pone.0094495`.

3. Gupta, S. B., Gilbert, R. L., Brady, A. R., Livingstone, S. J. & Evans, B. G. CD4 cell counts in adults with newly diagnosed HIV infection: results of surveillance in England and Wales, 1990-1998. *AIDS* **14**, 853–861 (2000). URL `https://journals.lww.com/aidsonline/Fulltext/2000/05050/CD4_cell_counts_in_adults_with_newly_diagnosed_HIV.12.aspx`.

4. CASCADE Collaboration. Differences in CD4 cell counts at seroconversion and decline among 5739 HIV-1–infected individuals with well-estimated dates of seroconversion. *J. Acquir. Immune Defic. Syndr.* **34**, 76–83 (2003). URL `https://journals.lww.com/jaids/Fulltext/2003/09010/Differences_in_CD4_Cell_Counts_at_Seroconversion.12.aspx`.
