# Supplementary material for: Transmitted HIV-1 is more virulent in heterosexual individuals than men-who-have-sex-with-men
Source: PLoS Pathog. 2022 Mar 10;18(3):e1010319. doi: 10.1371/journal.ppat.1010319 (PMC8912199; doi:10.1371/journal.ppat.1010319)
Supplement: S4 Table — The sample sizes (n) were reported, while cell counts were estimated using WebPlotDigitizer (https://automeris.io/WebPlotDigitizer). The last row represents estimates (see Methods) for the entire period 2006–12. (PDF) [file ppat.1010319.s004.pdf]

S4 Table. Early median CD4 cell counts in infected MSM and HET from China [1]. The sample sizes (n) were reported, while cell counts were estimated using WebPlotDigitizer ($https://automeris.io/WebPlotDigitizer$). The last row represents estimates (see Methods) for the entire period 2006-12.

Year	MSM		HET	
	Cell counts ($cells/\mu L$)	Sample size (n)	Cell counts ($cells/\mu L$)	Sample size (n)
2006	315	244	230	3,305
2007	345	672	249	7,251
2008	362	2,025	266	12,378
2009	364	3,624	265	18,208
2010	368	5,648	267	24,425
2011	370	9,316	271	35,211
2012	371	13,748	281	42,653
Average	368 ± 222 (SD†)	35,277	270 ± 260 (SD†)	143,431

†For the total population that contributed to the CD4 count data in China, consisting of MSM, HET and other transmission categories, during $2006 - 12$, the IQR was $130 - 454$ $cells/\mu L$. Using this, we obtained SD $= 240$ $cells/\mu L$ (see Methods). To calculate the SDs for MSM and HET, we recall that in the USA [2] the SDs corresponding to the CD4 counts from MSM, HET and the total population were 243, 284 and 262 $cells/\mu L$, respectively. Assuming that the ratios between SDs remain the same in China as well, we evaluated the SD for MSM to be 222 and HET to be 260 $cells/\mu L$ in China.

References

1. Tang, H. *et al.* Baseline CD4 cell counts of newly diagnosed HIV cases in China: 2006–2012. *PLoS ONE* **9**, e96098 (2014). URL `https://doi.org/10.1371/journal.pone.0096098`.
2. Robertson, M. M., Braunstein, S. L., Hoover, D. R., Li, S. & Nash, D. Estimates of the time from seroconversion to ART initiation among people newly diagnosed with HIV from 2006 to 2015, New York City. *Clin. Infect. Dis.* **71**, e308–e315 (2019). URL `https://doi.org/10.1093/cid/ciz1178`.
