# Supplementary material for: Transmitted HIV-1 is more virulent in heterosexual individuals than men-who-have-sex-with-men
Source: PLoS Pathog. 2022 Mar 10;18(3):e1010319. doi: 10.1371/journal.ppat.1010319 (PMC8912199; doi:10.1371/journal.ppat.1010319)
Supplement: S5 Table — Data from HIV/AIDS surveillance in Europe during 2009–2018 is used [27]. The cell counts were estimated using WebPlotDigitizer (https://automeris.io/WebPlotDigitizer). The median ages, where available, are provided. The last row provides the mean cell counts (with SDs) and total numbers of MSM, HET men and women, respectively, estimated as in Methods. (PDF) [file ppat.1010319.s005.pdf]

S5 Table. Early median CD4 cell counts in infected adults from EU/EEA. Data from HIV/AIDS surveillance in Europe during 2009-2018 is used [1]. The cell counts were estimated using WebPlotDigitizer ($https://automeris.io/WebPlotDigitizer$). The median ages, where available, are provided. The last row provides the mean cell counts (with SDs) and total numbers of MSM, HET men and women, respectively, estimated as in Methods.

Year	No. of diagnoses*		Fraction with CD4 counts††	Median age		Median CD4 count ($cells/\mu L$)		
	MSM	HET		MSM	HET	MSM	HET men	HET women
2010	10,348	11,693	0.591	-	-	420	261	312
2011	10,411	11,011	0.561	-	-	422	260	317
2012	11,238	10,782	0.553	-	-	436	270	319
2013	11,553	10,187	0.607	-	-	443	271	339
2014	11,821	10,049	0.614	-	-	450	281	346
2015	11,484	9,267	0.747	-	-	454	284	345
2016	10,508	9,003	0.673	34	39	440	274	351
2017	9,723	8,518	0.717	34	39	434	268	349
2018	8,049	7,267	0.695	36	41	423	262	340
Total	$n_{MSM} = 60,659$, $n_{HETM} = 27,822$, $n_{HETW} = 27,576$**					$(437 \pm 242)^‡$	$(270 \pm 258)^‡$	$(335 \pm 258)^‡$

*The number of diagnoses in each EU/EEA country during 2009-18 was available for MSM and HET [1], using which we estimated these numbers.
††Available in the annual surveillance reports. The number was unavailable for 2009.
**The numbers of HET men and women diagnosed from the 21 EU/EEA countries in 2018 were available in the 2019 annual report [1]. Using these numbers, we calculated the fractions of HET men and women and assumed that they remained the same during 2010-18. $n_{HET} = n_{HETmen} + n_{HETwomen} = 55,398$. (There were a small number of transgenders among HET in column 3, but the CD4 counts were reported only for HET men and women.)
‡SDs were not reported in the original study. We therefore used the SDs from the European population [2], mentioned in Tables 1 and S3 (see Methods). We also assumed HET men and women to have the same SD as that of the HET population.

References

1. European Centre for Disease Prevention and Control. *HIV/AIDS surveillance in Europe 2019* (HIV/AIDS surveillance in Europe 2009 - 2018 data). URL `https://www.ecdc.europa.eu/en/publications-data/hivaids-surveillance-europe-2019-2018-data`. [Online; accessed 28-December-2021].
2. Frentz, D. *et al.* Patterns of transmitted HIV drug resistance in Europe vary by risk group. *PLoS ONE* **9**, e94495 (2014). URL `https://doi.org/10.1371/journal.pone.0094495`.
