# Supplementary material for: Transmitted HIV-1 is more virulent in heterosexual individuals than men-who-have-sex-with-men
Source: PLoS Pathog. 2022 Mar 10;18(3):e1010319. doi: 10.1371/journal.ppat.1010319 (PMC8912199; doi:10.1371/journal.ppat.1010319)
Supplement: S6 Table — Mean CD4 counts in healthy adults from different population groups which define baseline counts for estimating the relative reduction in early cell count following HIV-1 infection. Sample sizes are in brackets. SD is standard deviation. (PDF) [file ppat.1010319.s006.pdf]

S6 Table. CD4 T cell counts in healthy adults. Mean CD4 counts in healthy adults from different population groups which define baseline counts for estimating the relative reduction in early cell count following HIV-1 infection. Sample sizes are in brackets. SD is standard deviation.

Region	Category (sample size, n)	Cell counts ($cells/\mu L$)	Other details
China (Hong Kong [1] & Shanghai [2])	Men (Hong Kong: 78 & Shanghai: 377)	725 (combined)	SD = 258 (Hong Kong) & 256 (Shanghai)
	Women (Hong Kong: 130 & Shanghai: 237)	724 (combined)	SD = 254 (Hong Kong) & 255 (Shanghai)
	HET (822)	725	SD = 255 (Shanghai)
Sub-Saharan Africa (from Tanzania) [3]	Men* (42)	666	SD = 247
	Women* (60)	802	SD = 250
	HET (102)	746	SD = 257‡‡
USA [4]	Men** (33)	921	SD = 188
	Women (67)	1041	SD = 340
	HET** (100)	1001	SD = 305
UK [5]	HET men† (50)	840	SD = 285
	HET women (50)	1050	SD = 377
	HET‡ (100)	945	We used the SD for women.
	MSM†,‡ (100)	800	SD = 324
EU/EEA (& Europe) & Australia (from Italy [6])	General population or HET†† (965)	941	SDs were not reported. We used the SDs from the UK study (see Methods).
	Men†† (532)	902	
	Women (436)	989	

* $P = 8 \times 10^{-3}$ for the comparison between men and women (from the original study).
** $P = 0.04$ for the comparison between healthy men and HET from USA.
† $P = 0.22$ for the comparison between MSM and HET men from UK.
‡ $P = 6.6 \times 10^{-4}$ for the comparison between healthy MSM and HET from UK, using SD (= 305) for HET from USA. Using SD (=377) corresponding to HET women from UK instead yielded $P = 2 \times 10^{-3}$.
†† $P = 0.018$ using the SDs from UK.
‡‡ For our calculation involving the EU/EEA 2010-18 population (footnote in S3 Table), we used the SD (=377) for the HET from Europe/UK.

References

1. Kam, K. M. *et al.* Lymphocyte subpopulation reference ranges for monitoring human immunodeficiency virus-infected Chinese adults. *Clin. Vaccine Immunol.* **3**, 326–330 (1996). URL `https://cvi.asm.org/content/3/3/326`.

2. Jiang, W. *et al.* Normal values for CD4 and CD8 lymphocyte subsets in healthy Chinese adults from Shanghai. *Clin. Vaccine Immunol.* **11**, 811–813 (2004). URL `https://cvi.asm.org/content/11/4/811`.

3. Ngowi, B. J., Mfinanga, S. G., Bruun, J. N. & Morkve, O. Immunohaematological reference values in human immunodeficiency virus-negative adolescent and adults in rural northern Tanzania. *BMC Infect. Dis.* **9**, 1 (2009). URL `https://doi.org/10.1186/1471-2334-9-1`.

4. Valiathan, R. *et al.* Reference ranges of lymphocyte subsets in healthy adults and adolescents with special mention of T cell maturation subsets in adults of South Florida. *Immunobiology* **219**, 487–496 (2014). URL `https://doi.org/10.1016/j.imbio.2014.02.010`.

5. Bofill, M. *et al.* Laboratory control values for CD4 and CD8 T lymphocytes. Implications for HIV-1 diagnosis. *Clin. Exp. Immunol.* **88**, 243–252 (1992). URL https://doi.org/10.1111/j.1365-2249.1992.tb03068.x.

6. Santagostino, A. *et al.* An Italian national multicenter study for the definition of a reference ranges for normal values of peripheral blood lymphocyte subsets in healthy adults. *Haematologica* **84**, 499–504 (1999). URL https://pubmed.ncbi.nlm.nih.gov/10366792/.
