# Supplementary material for: Transmitted HIV-1 is more virulent in heterosexual individuals than men-who-have-sex-with-men
Source: PLoS Pathog. 2022 Mar 10;18(3):e1010319. doi: 10.1371/journal.ppat.1010319 (PMC8912199; doi:10.1371/journal.ppat.1010319)
Supplement: S7 Table — This list includes all the populations we considered in the study. Where available, populations are divided into age-groups in addition to their combined set, as with the CASCADE 1979–00 datasets. For the continuous variables, the mean values are given where available. The diagnosis delay is expressed in years, whereas the viral load (SPVL) in log10 copies/mL. A diagnosis delay of 0.25 years is used for seroconversion. Details regarding the estimation of age are given in the text. The rows titled CASCADE 2003–05, 2006–09, and 2003–09 contain the groups that were mentioned in detail in Table 2 in the context of viral load data. The last four rows are created using the information on age in S5 Table. (PDF) [file ppat.1010319.s007.pdf]

S7 Table. The complete list of categorical and continuous variables. This list includes all the populations we considered in the study. Where available, populations are divided into age-groups in addition to their combined set, as with the CASCADE 1979-00 datasets. For the continuous variables, the mean values are given where available. The diagnosis delay is expressed in years, whereas the viral load (SPVL) in $\log_{10}$ copies/mL. A diagnosis delay of 0.25 years is used for seroconversion. Details regarding the estimation of age are given in the text. The rows titled CASCADE 2003-05, 2006-09, and 2003-09 contain the groups that were mentioned in detail in Table 2 in the context of viral load data. The last four rows are created using the information on age in S5 Table.

Cohort	Sex	Risk group	$R_{T/F}$ (%)	Age	Diagnosis delay	Subtype*	Ethnicity*	Viral load
CASCADE 1979-00	male	MSM	40	27.8	0.25	B	Caucasian	NA
	male	MSM	46.2	52.2	0.25	B	Caucasian	NA
	male	HET	46.4	27.8	0.25	Mixed (HET EU)	Mixed (HET EU)	NA
	male	HET	52.4	52.2	0.25	Mixed (HET EU)	Mixed (HET EU)	NA
	female	HET	46.4	22.7	0.25	Mixed (HET EU)	Mixed (HET EU)	NA
	female	HET	51.8	57.3	0.25	Mixed (HET EU)	Mixed (HET EU)	NA
	male	MSM	40.7	30.9	0.25	B	Caucasian	NA
	male	HET	47.2	30.9	0.25	Mixed (HET EU)	Mixed (HET EU)	NA
	female	HET	47.1	27	0.25	Mixed (HET EU)	Mixed (HET EU)	NA
	NA	HET	47.1	28.8	0.25	Mixed (HET EU)	Mixed (HET EU)	NA
CASCADE 2003-05	male	MSM	45.3	NA	0.25	B	Caucasian	4.46
	NA	HET	52.5	NA	0.25	Mixed (HET EU)	Mixed (HET EU)	4.55
CASCADE 2006-09	male	MSM	44	NA	0.25	B	Caucasian	4.4
	NA	HET	55.5	NA	0.25	Mixed (HET EU)	Mixed (HET EU)	4.4
CASCADE 2003-09	male	MSM	44.6	NA	0.25	B	Caucasian	4.43
	NA	HET	53.7	NA	0.25	Mixed (HET EU)	Mixed (HET EU)	4.49
US 2006-15†	male	MSM	51	21	0.25	B	Mixed (US)	NA
	NA	HET	64.7	21	0.25	B	Mixed (US)	NA
	male	MSM	73.7	36.1	4.05	B	Mixed (US)	NA
	NA	HET	85.8	36.1	5.4	B	Mixed (US)	NA
Europe 2002-07	male	MSM	67.8	36.1	NA	B	Caucasian	4.86
	NA	HET	88.8	37.7	NA	Mixed (HET EU)	Mixed (HET EU)	4.76
UK 1990-98††	male	MSM	78.2	33	NA	B	Caucasian	NA
	NA	HET	96	32	NA	Mixed (HET EU)	Mixed (HET EU)	NA
China 2006-12	male	MSM	68	NA	NA	Mixed (MSM CH)	Mixed (CH)	NA
	NA	HET	86.7	NA	NA	Mixed (HET CH)	Mixed (CH)	NA
EUEEA 2010-18	male	MSM	66.2	NA	NA	B	Caucasian	NA
	male	HET	90	NA	NA	Mixed (HET EU)	Mixed (HET EU)	NA
	female	HET	82.9	NA	NA	Mixed (HET EU)	Mixed (HET EU)	NA
	NA	HET	86.2	NA	NA	Mixed (HET EU)	Mixed (HET EU)	NA
EUEEA 2016-17	male	MSM	66.3	34	NA	B	Caucasian	NA
	NA	HET	85.5	39	NA	Mixed (HET EU)	Mixed (HET EU)	NA
EUEEA 2018	male	MSM	68.2	36	NA	B	Caucasian	NA
	NA	HET	86.8	41	NA	Mixed (HET EU)	Mixed (HET EU)	NA

*HIV-1 infected European HET and Chinese individuals contain a mixture of subtypes, with two or more subtypes accounting for a substantial fraction of the infections. Further, subtype compositions in MSM and HET in China are different. We denote the mixed subtypes in the different populations using Mixed (HET EU), Mixed (HET CH), and Mixed (MSM CH), respectively. Similarly, we denote the mixed ethnicities in China, in the US, and in European HET using Mixed (CH), Mixed (US), and Mixed (HET EU), respectively.

†For the US population at diagnosis, mean age is calculated using the reported age distribution [1] and assuming the mean age in the group aged>50 to be 60.

††Age is measured as the mid-value during the study period.

References

1. Robertson, M. M., Braunstein, S. L., Hoover, D. R., Li, S. & Nash, D. Estimates of the time from seroconversion to ART initiation among people newly diagnosed with HIV from 2006 to 2015, New York City. *Clin. Infect. Dis.* **71**, e308–e315 (2019). URL `https://doi.org/10.1093/cid/ciz1178`.
