# Supplementary material for: Transmitted HIV-1 is more virulent in heterosexual individuals than men-who-have-sex-with-men
Source: PLoS Pathog. 2022 Mar 10;18(3):e1010319. doi: 10.1371/journal.ppat.1010319 (PMC8912199; doi:10.1371/journal.ppat.1010319)
Supplement: S8 Table — The percentages of individuals found to be associated with transmission clusters in MSM and HET in several studies are collated. In the second column, the numbers in parantheses indicate sample sizes examined. Where available, P values and the largest cluster sizes are indicated as other details. (PDF) [file ppat.1010319.s008.pdf]

S8 Table. Association of MSM and HET with transmission clusters. The percentages of individuals found to be associated with transmission clusters in MSM and HET in several studies are collated. In the second column, the numbers in parantheses indicate sample sizes examined. Where available, P values and the largest cluster sizes are indicated as other details.

Region (Study period)	Group (n)	Associated with clusters (%)	Other details
Japan (2004 – 11) [1]	MSM (261)	38.3	Clusters are also linked to China.
	HET (103)	8.7	
Austria (2008 – 14) [2]	MSM (122)	57.4	$P < 0.001$
	HET (115)	32.2	
Belgium (2013 – 15) [3]	MSM (698)	57.7	$P < 0.001$. Cluster size ≥ 3. The largest cluster is sized 94, consisting of 68 MSM and 15 HET.
	HET (508)	22.4	
Cyprus (1986 – 2012) [4]	MSM (164)	36.6	Cluster size ≥ 3. The two largest clusters are sized 13, having 11 – 12 MSM.
	HET (129)	2.3	
France (1999 – 2014) [5]	MSM (636)	32.1	$P < 0.01$. Cluster size ≥ 3. The largest cluster, sized 41, has 39 MSM and a HET.
	HET (267)	3.8	
Germany (1999 – 2016) [6]	MSM (1,448)	27.3	
	HET (622)	18.2	
Netherlands (1981 – 2011) [7]	MSM (4,288)	51.2	Cluster size ≥ 10.
	HET (849)	55.8	
Spain (2004 – 14) [8]	MSM (637)	–	8 of 12 clusters sized ≥ 10 consist only of MSM. The largest cluster has a size of 111, and is composed only of MSM.
	HET (204)	–	
USA (2001 – 15) [9]	MSM (1,597)	37.4	The largest cluster has a size of 39, and has only MSM.
	HET (953)	28.3	
Canada and 9 European countries (1981 – 2011) [10]	MSM (4,980)	51.4	The 9 countries are Italy, Greece, Netherlands, Germany, France, UK, Norway, Austria, and Spain. $P < 0.001$.
	Bisexual men (2,087)	27.7	

References

1. Kondo, M. *et al.* Emergence in Japan of an HIV-1 variant associated with transmission among men who have sex with men (MSM) in China: first indication of the international dissemination of the Chinese MSM lineage. *J. Virol.* **87**, 5351–5361 (2013). URL `https://jvi.asm.org/content/87/10/5351`.

2. Hoenigl, M. *et al.* Characterization of HIV transmission in South-east Austria. *PLoS ONE* **11**, e0151478 (2016). URL `https://doi.org/10.1371/journal.pone.0151478`.

3. Verhofstede, C. *et al.* Phylogenetic analysis of the Belgian HIV-1 epidemic reveals that local transmission is almost exclusively driven by men having sex with men despite presence of large African migrant communities. *Infect. Genet. Evol.* **61**, 36–44 (2018). URL `https://doi.org/10.1016/j.meegid.2018.03.002`.

4. Pineda-Peña, A. *et al.* HIV-1 infection in Cyprus, the Eastern Mediterranean European frontier: a densely sampled transmission dynamics analysis from 1986 to 2012. *Sci. Rep.* **8**, 1702 (2018). URL `https://doi.org/10.1038/s41598-017-19080-5`.
5. Chaillon, A. *et al.* Spatiotemporal dynamics of HIV-1 transmission in France (1999-2014) and impact of targeted prevention strategies. *Retrovirology* **14**, 15 (2017). URL `https://doi.org/10.1186/s12977-017-0339-4`.
6. Stecher, M. *et al.* Molecular epidemiology of the HIV epidemic in three German metropolitan regions – Cologne/Bonn, Munich and Hannover, 1999–2016. *Sci. Rep.* **8**, 6799 (2018). URL `https://doi.org/10.1038/s41598-018-25004-8`.
7. Bezemer, D. *et al.* Dispersion of the HIV-1 epidemic in men who have sex with men in the Netherlands: a combined mathematical model and phylogenetic analysis. *PLoS Med.* **12**, e1001898 (2015). URL `https://doi.org/10.1371/journal.pmed.1001898`.
8. Patino-Galindo, J. A. *et al.* The molecular epidemiology of HIV-1 in the Comunidad Valenciana (Spain): analysis of transmission clusters. *Sci. Rep.* **7**, 11584 (2017). URL `https://doi.org/10.1038/s41598-017-10286-1`.
9. Dennis, A. M. *et al.* HIV-1 transmission clustering and phylodynamics highlight the important role of young men who have sex with men. *AIDS Res. Hum. Retroviruses* **34**, 879–88 (2018). URL `https://doi.org/10.1089/aid.2018.0039`.
10. Paraskevis, D. *et al.* HIV-1 molecular transmission clusters in nine European countries and Canada: association with demographic and clinical factors. *BMC Med.* **17**, 4 (2019). URL `https://doi.org/10.1186/s12916-018-1241-1`.
