# Supplementary material for: Transmitted HIV-1 is more virulent in heterosexual individuals than men-who-have-sex-with-men
Source: PLoS Pathog. 2022 Mar 10;18(3):e1010319. doi: 10.1371/journal.ppat.1010319 (PMC8912199; doi:10.1371/journal.ppat.1010319)
Supplement: Attachment — Submitted filename: Response to Reviewers Comments.pdf [file ppat.1010319.s009.pdf]

RESPONSE TO REVIEWERS' COMMENTS

on our manuscript with the tracking number PPATHOGENS-D-21-01951 entitled, "Transmitted HIV-1 is more virulent in heterosexual individuals than men-who-have-sex-with-men"

We are grateful to both the reviewers for their overall positive assessment of our manuscript and for their insightful observations and comments. Addressing their comments, we believe, has greatly enriched and improved our manuscript. Below, we present our responses to their comments. The comments are reproduced in blue and are followed by our responses. Text reproduced from the manuscript shows changes made in red.

Part I - Summary

Please use this section to discuss strengths/weaknesses of study, novelty/significance, general execution and scholarship.

Reviewer #1: This manuscript investigates the possible effect of within risk group transmission on the HIV virulence. This is a previously looked at association, which the authors appropriately cite, and here they investigate a large collection of data. The authors find evidence that suggest that HIV spread among MSM vs HSX differ such that MSM virus cause less severe infections than HSX virus.

Reviewer #2: This is an interesting manuscript that investigates differences in CD4 T cell count at the time of HIV infection diagnosis or seroconversion, as a measure of virulence, in men who have sex with men (MSM) and heterosexuals (HSX). The authors hypothesize that such differences are the result of differential selection bias during transmission and examine early CD4 T cell count measurements reported from 340,000 infected HSX individuals and MSM, across geographies, ethnicities and calendar year. The authors' conclusion is that, because MSM have higher CD4 counts at the time of diagnosis and seroconversion than HSX, this reflects the greater selection bias for more fit viruses during penile/vaginal sex compared to anal sex. Surprisingly, this increased "virulence" was not reflected in higher VL in HSX individuals vs MSM. This large meta-analysis is quite novel and yields results that support differences in viral pathogenicity developing over time in different risk populations.

Response: We thank the reviewers for these opening remarks succinctly summarizing our findings and highlighting the novelty of our study.

Part II – Major Issues: Key Experiments Required for Acceptance

Please use this section to detail the key new experiments or modifications of existing experiments that should be absolutely required to validate study conclusions.

Generally, there should be no more than 3 such required experiments or major modifications for a "Major Revision" recommendation. If more than 3 experiments are necessary to validate the study conclusions, then you are encouraged to recommend "Reject".

Reviewer #1: While the authors do address confounding issues, one issue stands out as more severe than the others, namely the fact that HSX are diagnosed significantly later than MSM (for sure in US and EU). The authors qualitatively and anecdotally comment on this, but do not attempt to quantitate this effect as they do with other potentially confounding factors. The later diagnosis would certainly bias the results to see HSX with lower CD counts and more advanced disease at time of diagnosis.

If the effect of within risk group transmission was an ongoing process (and there is little cross-transmission between MSM and HSX, as shown), then why is the difference in virulence not changing over time? The apparent lack of HSX-virus getting more virulent over time suggests that the difference in MSM and HSX virus is not an effect of a process but rather something that does not change, like the fact that HSX are diagnosed later (with a similar delay over time).

Even if MSM and HSX transmissions mostly happen within these risk groups there is transfer between them. Depending how frequent and along what type of network such transfer happens, different results are expected when measuring within the risk groups. Can the authors address this with some (compartmental?) modeling or at least discuss how that complicates detection of differences qualitatively, using previous research on the connections between risk groups? Could this explain why there are no time trends in virulence?

Response: The reviewer has raised several important points here and we have addressed them all in the revised version as follows.

The greater diagnosis delay in HET (we have replaced HSX by HET throughout, as suggested by the reviewer below) than MSM could indeed result in lower CD4 counts in HET at diagnosis than MSM, confounding the effect of risk groups. We had previously argued against this confounding effect qualitatively. We have now quantified it. The diagnosis delays in HET and MSM are known in the US. Also, the rate of CD4 decline following seroconversion have been estimated. Using the latter rate and the measured CD4 counts at diagnosis, the CD4 counts in one risk group (say MSM) can be projected (or extrapolated) to the time of diagnosis of the other group (HET), thus eliminating the confounding due to the differential diagnosis delay in the two groups. We mention this approach in the Methods (Page 22, line 440):

"The above projection technique could be used to estimate CD4 counts at other times post seroconversion too. We applied it to estimate the CD4 counts in HET at the time of diagnosis in MSM and in MSM at the time of diagnosis in HET. This allowed comparisons between the two groups without the confounding effects of different diagnosis delays."

We found when we did this that the late diagnosis in HET could only explain a part of the difference in early CD4 counts between MSM and HET. Furthermore, we could project the CD4 counts all the way to seroconversion, which is expected to occur at the same time in the two risk groups, thus eliminating the effect of diagnosis delay completely. Interestingly, the gap in the early CD4 counts ($R_{T/F}$ values) between the two groups widened at seroconversion compared to diagnosis, indicating that the effect of risk group was not an artefact of diagnosis delay. (The shrinking of the difference with time is because $R_{T/F}$ in both groups gradually increase and reach 100%. The gap thus gradually thins and eventually vanishes.) This effect was true also in European cohorts,

further substantiating our claim. We present this analysis in the revised manuscript (Page 16, line 248):

"[W]e quantified the effect of diagnosis delay on our estimates of the differences in $R_{T/F}$ between MSM and HET. In the US, the median diagnosis delays during 2006-15 were ~4 years and ~5.4 years for MSM and HET, respectively[30,56]. Using the CD4 counts at diagnosis and their subsequent decline rates[30,56], we projected the CD4 counts in MSM to 5.4 years post seroconversion (Methods), which yielded $R_{T/F}$ of 80.3%, substantially lower than that for HET of 85.8% at the same time (Table 1). Projecting the $R_{T/F}$ of HET to 4 years post seroconversion and comparing with MSM yielded similar conclusions. Thus, diagnosis delay could explain only a part of the differences in $R_{T/F}$ between MSM and HET at diagnosis. Indeed, as mentioned above, extrapolating all the way to seroconversion, thereby eliminating effects of diagnosis delay completely, did not eliminate the differences in $R_{T/F}$ between MSM and HET (Table 1). Rather, the difference was amplified ($R_{T/F}$ was 51% in MSM and 64.7% in HET; see Table 1). (Note that with time $R_{T/F}$ in both MSM and HET would approach 100%, shrinking the difference between them and explaining the amplified difference at seroconversion.) Similarly, after extrapolating to seroconversion, substantial differences (~20 percentage points) in $R_{T/F}$ persisted in the UK and EU/EEA cohorts as well, ruling out diagnosis delay as a major confounding factor."

We agree with the reviewer that virulence is expected to change with time. Measurements of CD4 counts at seroconversion over the years indicate that virulence increased with time (CD4 counts at seroconversion decreased) from the early 1980's to the early 2000's, following which the virulence seems to have largely stabilized (Pantasiz et al., Lancet HIV, 2014). This trend was seen in all the major risk groups. Four of the six datasets we examined in our study, which included all but 19000 of the 340000 patient datapoints we considered, were from the periods post the early 2000s, when substantial evolution of virulence has not been observed. Consequently, averaging the CD4 counts over the study durations, as we have done, appears reasonable. Yet, we do recognize that the evolutionary plateaus to which the CD4 counts have settled in MSM and HET are distinct and suggest distinct evolutionary pressures in the two groups. The origins of these differences remain unknown and would be important to elucidate in future studies. Nonetheless, the apparent absence of evolution is not due to some confounding/static effect such as diagnosis delay and appears to be a more fundamental property of HIV adaptation within human hosts. We have now added a discussion of these issues in the revised manuscript (Page 19, line 337):

"More fundamentally, whether HIV-1 is evolving in humans to be more or less virulent with time remains an unresolved question. Different studies have argued that the virulence is increasing[61], stable[62], or decreasing[44] with time. A more recent study that examined trends in CD4 counts at seroconversion in the CASCADE cohorts found that the counts declined from the early 1980s and reached a plateau around 2002, following which significant evolution was not evident[41]. The causes of these trends have remained elusive. In our present study, 4 of the 6 datasets we examined, which contained all but 13000 patients, belong to the post 2002 period (Table 1), where evolution of early CD4 counts is expected to be minimal, because of which we used counts averaged over the study periods in the respective datasets. An inference from our findings would thus be that the early CD4 counts in MSM and HET appear to have settled over the years to

distinct plateau values. How the differential selection bias at transmission in the two groups, together with other potential evolutionary pressures, may have resulted in the different plateaus is an interesting question to address. Complex nested models[63,64], which couple within-host and population-level evolution of the virus, would have to be developed, perhaps incorporating selection over multiple heritable traits, to help deduce the causes of the observed evolutionary trends."

Finally, we considered the issue of mixing between MSM and HET. We agree with the reviewer that depending on the extent of mixing and the nature of the underlying transmission networks, the effects within groups could be affected. Studies from Nordic states, where such mixing and the resulting transmission have been best followed, suggest that the chance of such mixing leading to the establishment of a sustained epidemic may be low. Indeed, where population densities are low, such as in Greenland, estimates are that 1 in 25 mixing events may lead to a sustained epidemic. This is partly because the transmission from such mixing is typically into small clusters that are likely to be isolated even within risk groups. Thus, homogenizing of the strains or large sweeps across populations due to such mixing appears a rare event. This is perhaps why HIV-1 subtypes remain quite distinct across risk groups (as we have shown in Fig. 1B.) Nonetheless, could the limited mixing explain the absence of time trends in virulence? As we mention above, time trends in virulence do exist, but seem to have diminished post the early 2000s, the reasons for which remain unknown. We examined available compartmental models to assess the effects of mixing on virulence. Presently, models are being developed to examine whether mixing between geographical regions (migration) among 'identical' hosts can explain the existence of the epidemic due to the 'same' virus in regions of heterogeneous population densities. These models are quite complex even without considering the evolution of virulence in identical hosts. Advancing them to include the evolution of virulence is yet to be accomplished. Subsequently, the models would have to consider different predominant strains circulating in distinct risk groups to assess the effect of mixing. While important and challenging, these endeavors are beyond the scope of the present study. We have introduced a separate paragraph discussing these issues and highlighting the challenges involved in developing suitable compartmental models for assessing the effects of mixing in the revised manuscript (Page 19, line 352):

"Another aspect that warrants further investigation is the influence of mixing between groups. Although evidence of minimal mixing between MSM and HET exists, including from the comprehensive data of subtype prevalences that we have collated as part of the present study, the effect that the limited mixing may have on the differences in early CD4 counts, and hence $R_{T/F}$, between the groups remains to be quantified. Specifically, how a strain circulating in MSM, for instance, would behave when transmitted to HET, due to mixing, and vice versa, would be interesting to examine. Detailed natural history of the HIV epidemic in select countries provides some insights[65,66]. If the population density is small, most transmission events may not lead to the establishment of an epidemic; indeed, one of approximately 25 transmission events is estimated to have resulted in a sustained epidemic in Greenland[65]. The intriguing notion of source-sink dynamics, developed first for ecological settings, has recently been proposed as relevant to natural HIV infection to assess how epidemics are sustained in geographically separated populations with diverse population densities[67]. Here, a region of high population density with a

sustained epidemic can ensure the sustenance of the epidemic in a region of low population density via migration. We envision such approaches being adapted to assess the effect of mixing between risk groups. We recognize though that such models will necessarily be more involved than models aimed at describing the effects of geographical heterogeneity in population density. In the latter case, the virus and host traits are identical. With risk groups and possibly different phenotypic T/F strains, viral evolution will have to be superimposed on a spatial epidemic model."

Reviewer #2: Overall, the results presented from this very large multi-cohort analysis do point to a greater impact of early HIV-1 infection on CD4 T cell counts in HSX individuals compared to MSM. Nevertheless, for most European countries (including the UK) there is a significant fraction of HIV-1 infections initiated by non-B subtype viruses in the HSX cohorts and while the authors point to data from the US, where subtype B is predominant in both groups, and a couple of papers suggesting that subtype B is more pathogenic, they do not test this formally using statistical analyses. Since for most cohorts the subtype composition is significantly different between MSM and HSX, this analysis should be performed.

Response: We thank the reviewer for this comment. We agree and have now performed regression analysis to ascertain the effect of subtypes on the difference in early CD4 count measurements between MSM and HET. We found that the effect was small (~2 percentage points in $R_{T/F}$, whereas the overall difference is ~18 percentage points). The difference due to the transmission bottleneck between risk groups is thus robust to the subtype differences in the groups. We present this finding in the revised manuscript (Page 16, line 268 in Results and Page 26, line 524 in the Methods):

"We quantified the effect of subtype on $R_{T/F}$ and found that a negligible portion (~2 percentage points) of the difference in $R_{T/F}$ between MSM and HET was attributable to subtype (Methods). We concluded therefore that subtype was not a factor confounding our inference above."

"To estimate the potential contribution from subtype, we extended the regression analysis to include subtype and applied it to the EU/EEA cohort, where subtypes differ between MSM and HET. We thus wrote $R_{T/F} = \beta_0 + \beta_1 X_1 + \beta_2 X_2 + \beta_3 X_3 + \beta_4 X_4$, where β_4 is the effect subtype on $R_{T/F}$ and X_4 is a categorical variable representing subtype. We set X_4=0 for subtype B, dominant in MSM, and X_4=1 for the collective of subtypes present in HET. The other terms were the same as in Eq. (1). We used the diagnosis delays of 3 years for MSM and 4.9 years for HET men[82] and, following the procedure above, estimated the CD4 counts at seroconversion to be 412 cells/μL in HET men ($R_{T/F}$ = 69.8%) and 561 cells/μL in MSM ($R_{T/F}$ = 48.6%). Using estimates of β_1 and β_3 above together with the latter estimates of $R_{T/F}$, we calculated β_4=2.2 percentage points, indicating a negligible effect of subtype on $R_{T/F}$."

Part III – Minor Issues: Editorial and Data Presentation Modifications

Please use this section for editorial suggestions as well as relatively minor modifications of existing data that would enhance clarity.

Reviewer #1: 1. The risk of MSM infection is greater than that of HSX; and the MSM bottleneck lets >1 virus through more often than in HSX (Leitner & Romero-Severson, Nat Microbiol 2018). Thus, the genetic bottleneck is less strict in MSM. How may that affect the issue studied here?

Response: We appreciate the issue raised by the reviewer. The larger number of transmitted strains in MSM is likely to have two key effects. First, it increases the chances of the infection getting established per sexual act. Because we are concerned about the CD4 counts post the establishment of infection, this effect is not critical to the present study. Second, it could increase the diversity of the viral strains present and increase the chances of immune escape. This too is not critical to our current study. The early CD4 counts are typically thought to be established before substantial adaptation of the virus to the host occurs. Further, the potentially greater evolution in MSM due to the larger number of strains should have increased the virulence in MSM over HET, an effect that is opposite to the present findings. Thus, accounting for this effect would only enhance the estimated difference between MSM and HET studied here. We discuss this aspects briefly in the revised manuscript (Page 20, line 370):

"The less stringent transmission bottleneck associated with anal transmission than penile-vaginal transmission results not only in less virulent T/F strains but may also allow a greater number of T/F viruses to establish infection in MSM than HET[68]. (Evidence that the number is not greater also exists[3].) The implications of the greater number of T/F strains remain to be elucidated. We speculate that its effect on early CD4 count decline is likely to be minimal because the latter represents a rapidly established balance between viral replication and immune control before much viral adaptation to the host. Thus, we expect our estimates of $R_{T/F}$ to remain robust to the variations in the number of T/F virions. On the other hand, the subsequent decline of CD4 counts leading to AIDS may be affected by these variations. A greater number of T/F virions may imply greater genomic diversity, which may allow easier immune escape, including via recombination[69,70], and expedite CD4 decline. Indeed, in some studies, MSM have been observed to exhibit faster CD4 decline despite higher initial CD4 counts than HET[29,40]."

Reviewer #1: 2. HET is a more common abbreviation of heterosexual (transmission) than HSX.

Response: We thank the reviewer for pointing this out and have now replaced HSX by HET throughout.

Reviewer #2: 1. Despite the fact that the authors show in Table S6 that, with the exception of China, healthy women generally exhibit higher CD4 T cell counts than men, this is not fully developed in the paper. For the most part men and women are lumped together in the HSX category – rather than being considered separately where the data are available. A direct comparison of MSM with HSX men would seem to be a much more direct comparison and likely would reveal an even greater impact on CD4 counts and R values between the two groups. This data is presented for EU/EAA 2010-18 at diagnosis and CASCADE seroconverters in Table 1 but is not developed or discussed in detail in the paper.

Response: We agree and have now added direct comparisons between MSM and HET men. Indeed, as the reviewer expects, the differences are greater than between MSM and HET, highlighting further the effect of the transmission bottleneck. We present these new comparisons in the revised manuscript (Page 10, line 161):

"In some studies, data was available separately for HET men and women, allowing a comparison between HET men and MSM, thus eliminating potential confounding effects of sex (Table 1). In EU/EEA, during 2010-18, $R_{T/F}$ in HET men was 90%, much higher than the 82.9% in HET women, indicating that the difference between HET and MSM was amplified upon eliminating the effect of sex. We recall that $R_{T/F}$ was 66.2% in MSM during the same period, significantly smaller than HET men ($P<10^{-4}$; d_s=0.63) and HET women ($P<10^{-4}$; d_s=0.20). Among the seroconverters in Europe and Australia, in those aged below 40 years, $R_{T/F}$ in HET men was 46.4%, higher than the 40% in MSM (P=0.01; d_s=0.12). The difference was similar in those aged above 40 years; $R_{T/F}$ was 52.4% in HET men and 46.2% in MSM (P=0.073; d_s=0.16). Thus, in these comparisons too, where the effects of sex, age, and diagnosis delay were eliminated, HET had a consistently higher $R_{T/F}$ than MSM."

Reviewer #2: 2. The significance of the per parasite pathogenicity analysis is not clear. In many ways the pathogenicity values derived in this section seem to be simply restating in a somewhat redundant way the difference in R between the two groups – a better rationale for defining this term as an independent value is needed.

Response: We have now provided a better rationale for the per parasite pathogenicity. The reason the present calculation appears to be a restatement of the $R_{T/F}$ values is because the set-point viral loads are very similar between MSM and HET. Had they been different, the calculation of per parasite pathogenicity would have been more involved. We now perform this more involved calculation and show that the estimates are similar to those we obtain by simply comparing $R_{T/F}$ values because of the similarity in the viral loads. We present these details in a separate section in the Methods (Page 23, line 458):

"**Estimation of per parasite pathogenicity.** To estimate the relative per parasite pathogenicity, $P_{T/F}$, we adopted the procedure developed earlier[10,11]. Accordingly, one would deduce the dependence of $R_{T/F}$ on SPVL, or tolerance, in one group. Using the dependence, the $R_{T/F}$ of the group at the SPVL in the other group would be predicted. The deviation of the predicted $R_{T/F}$ from that measured in the other group would yield the relative $P_{T/F}$. This procedure would thus quantify the effect of the T/F strain on $R_{T/F}$ beyond that expected from SPVL. As the SPVL were similar in MSM and HET, it followed that the difference in the measured $R_{T/F}$ between the groups would yield accurate estimates of $P_{T/F}$. We ascertained this by also following the above procedure explicitly. We estimated the tolerance of MSM as $\alpha = \frac{R_{T/F}^{MSM}}{\left(SPVL^{MSM}\right)^2}$, assuming that CD4 decline (and hence $R_{T/F}$) was proportional to the square of SPVL[10,11]. Assuming a linear dependence instead did not alter our conclusions. The per-parasite pathogenicity[11] of HET relative to MSM

was then $P_{T/F} = \frac{R_{T/F}^{HET}}{\left(SPVL^{HET}\right)^2} - \alpha$. We quantified the percentage contribution of $P_{T/F}$ to $R_{T/F}$ as

$$P_{T/F} \times \frac{\left(SPVL^{HET}\right)^2}{R_{T/F}^{HET}} \times 100 = \frac{R_{T/F}^{HET} - R_{T/F}^{MSM} \times \left(\frac{SPVL^{HET}}{SPVL^{MSM}}\right)^2}{R_{T/F}^{HET}} \times 100$$

. We found that the contributions varied from ~10-25%, which were similar to the values obtained by assuming that the SPVL were identical in the two groups (Table. 2)."

Reviewer #2: 3. The consistency of the results from China with those for other countries is somewhat surprising given that many of the initial heterosexual cases likely resulted from contaminated blood supplies. This is not discussed in the text.

Response: It is true that the early heterosexual cases were from contaminated blood supplies. However, HIV-1 testing before blood donation became mandatory in China in the mid-90s. The dataset from China that we have considered is from 2006-12. Thus, contaminated blood supplies may not affect our present comparisons. We now mention this in Table 1 (footnote):

"We ruled out any bias in the results from China due to contaminated blood supplies, as HIV-1 testing before blood donation became mandatory in the mid-1990s[33,34]."
